# Novel Compound Heterozygous *BBS2* and Homozygous *MKKS* Variants Detected in Chinese Families with Bardet–Biedl Syndrome

**DOI:** 10.1155/2021/6751857

**Published:** 2021-01-06

**Authors:** Li Huang, Limei Sun, Zhirong Wang, Songshan Li, Chonglin Chen, Xiaoling Luo, Xiaoyan Ding

**Affiliations:** State Key Laboratory of Ophthalmology, Zhongshan Ophthalmic Center, Sun Yat-Sen University, 54 Xianlie Road, Guangzhou 510060, China

## Abstract

**Background:**

Bardet–Biedl syndrome (BBS) is a rare multisystem developmental disorder. In this study, we report the genetic causes and clinical manifestations in two Chinese families with BBS.

**Materials and Methods:**

Two families were recruited in this study. Family A was a four-generation family with four affected and 15 unaffected members participating in the study, and family B was a consanguineous family with one affected and three unaffected members participating. Whole exome sequencing was performed in the two families, followed by a multistep bioinformatics analysis. Sanger sequencing was used to verify the variants and to perform a segregation analysis. Comprehensive ocular and systemic examinations were also conducted.

**Results:**

Novel compound heterozygous variants c.235T > G (p.T79P) and c.534 + 1G > T were detected in the *BBS2* gene in family A, and known homozygous variant c.748G > A (p.G250R) was detected in the *MKKS* gene in family B. Both families presented with retinitis pigmentosa; however, except for polydactyly, all other systemic manifestations were different. All of the affected family members in family A were overweight with a high body mass index (range from 26.5 to 41.9) and high blood pressure. Family A also presented with a delay in the onset of secondary sex characteristics and genital anomalies, while other systemic abnormalities were absent in family B.

**Conclusions:**

This study presents one family with two novel *BBS2* variants, expanding the variant spectrum of BBS, and one family with a known homozygous *MKKS* variant. The different phenotypes seen between the families with *BBS2* and *MKKS* variants will contribute to the literature and our overall understanding of BBS.

## 1. Introduction

Bardet–Biedl syndrome (BBS; MIM: 209900) is a rare multisystem developmental disorder with a prevalence of 1 : 13,500 to 1 : 160,000 [1, 2]. The characteristics of BBS include rod-cone dystrophy, truncal obesity, postaxial polydactyly, cognitive impairment, male hypogonadotropic hypogonadism, complex female genitourinary malformations, and renal abnormalities [3].

According to the Retinal Information Network (RetNet; https://sph.uth.edu/retnet/sum-dis.htm),a total of 26 genes, including *ADIPOR1* (MIM: 607945) [4], *ARL6* (MIM: 608845) [5], *BBIP1* (MIM: 613605) [6], *BBS1* (MIM: 209901) [7], *BBS2* (MIM: 615981) [8], *BBS4* (MIM: 615982) [9], *BBS5* (MIM: 603650) [10], *BBS7* (MIM: 607590) [11], *BBS9* (MIM: 607968) [12], *BBS10* (MIM: 615987) [13], *BBS12* (MIM: 615989) [14], *C8orf37* (MIM: 614477) [15], *CEP290* (MIM: 610142) [16], *IFT172* (MIM: 607386) [17], *IFT27* (MIM: 615870) [18], *INPP5E* (MIM: 613037) [19], *KCNJ13* (MIM: 603208) [20], *LZTFL1* (MIM: 606568) [21], *MKKS* (MIM: 604896) [22], *MKS1* (MIM: 609883) [23], *NPHP1* (MIM: 607100) [24], *SDCCAG8* (MIM: 613524) [25], *TRIM32* (MIM: 602290) [26], *TTC8* (MIM: 608132) [27], *SCLT1* (MIM: 611399), and *CEP164* (MIM: 614848) [28], have currently been reported to cause BBS.

In this study, we report on two Chinese families with BBS, including their gene variants and clinical manifestations.

## 2. Patients and Methods

Two families were recruited from the Zhongshan Ophthalmic Center (Guangzhou, China). Nineteen participants, including four affected and 15 unaffected, were included from four-generation family A. Four participants, including one affected and three unaffected, were recruited from consanguineous family B. Written informed consent was obtained from each participating individual or a guardian prior to the study. This study was approved by the Institutional Review Board of the Zhongshan Ophthalmic Center. Genomic DNA was prepared from venous leukocytes for all 23 family members using a previously described method [29]. The diagnosis for BBS was made according to the criteria for an improved diagnosis of BBS [30]. All research adhered to the tenets of the Declaration of Helsinki.

Whole exome sequencing (WES) was performed in six family members in family A: III : 4, III : 5, IV : 1, IV : 5, IV : 8, and IV : 10 and one family member in family B: IV : 2. The WES was performed using the Illumina MiSeq platform (Illumina, Madison, WI, USA) and average sequencing depth was set to 100-fold. Strand NGS software version 2.0 (Strand Scientific Intelligence Inc., LA, USA) was used to set the sequencing reads to University of California Santa Cruz hg19. The Human Gene Mutation Database (HGMD; http://www.hgmd.cf.ac.uk/ac/index.php), The Exome Aggregation Consortium (ExAC; http://exac.broadinstitute.org/), and dbSNP (https://www.ncbi.nlm.nih.gov/snp/) were taken into account to determine the frequency of the variants. Online algorithms (SIFT, PolyPhen-2, Mutation Taster, and PROVEAN) were used to estimate the pathogenicity of the mutated genes, and the Human Splicing Finder (HSF; http://umd.be/HSF3/) was used to study pre-mRNA splicing sites [31]. Sanger sequencing was used to verify the identified variants, and a segregation analysis was performed in the available family members.

Detailed clinical data was collected, including age, gender, height, weight, blood pressure, and heart rate. Visual acuity was measured with a Snellen visual chart. The eyes were examined with a hand-held slit lamp. Fundus photography was taken with a hand-held nonmydriatic digital fundus camera (Optomed Oy, Oulu, Finland). The hearing test was performed using a hand-held device (Interacoustics, Middelfart, Denmark). Ultrasound (GE Healthcare, Chicago, USA) was performed on the heart, kidney, and genitals. Full field electroretinogram (RETIport, Roland Consult, Brandenburg, Germany) was performed in the probands of families A and B.

## 3. Results

### 3.1. Genetic Information

**Figure 1:**
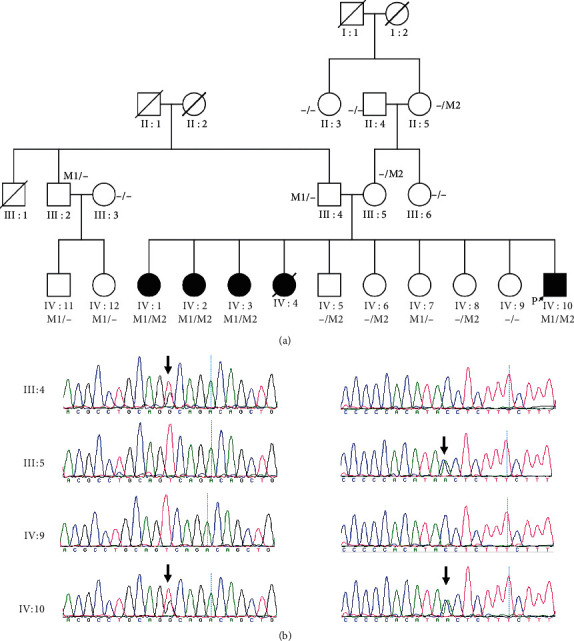
The pedigree and DNA sequencing of family (a) A. The pedigree of family A: M1 stands for variant c.235T > G (p.T79P) in the *BBS2* gene, and M2 stands for variant c.534 + 1G > T in the *BBS2* gene. (b) The Sanger sequencing of this family. The left column is the sequencing of variant c.235T > G, and the right column is the sequencing of variant c.534 + 1G > T. The arrows indicate where the variant is.

**Figure 2:**
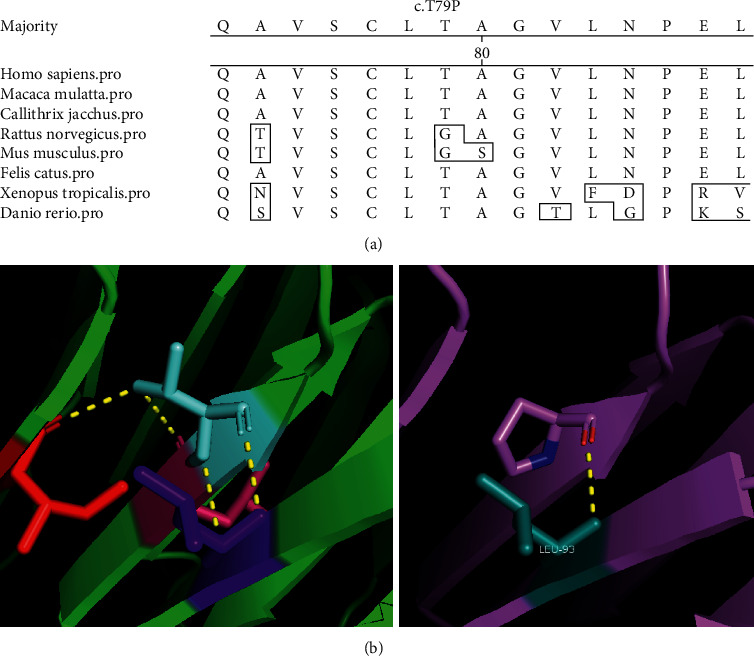
Conservation analysis and protein structure changes with variant p.T79P in the *BBS2* gene. (a) The threonine located at position 79 is conserved residue among vertebrates. (b) The hydrophilic threonine was substituted to hydrophilic proline, causing the protein structure change.

**Figure 3:**
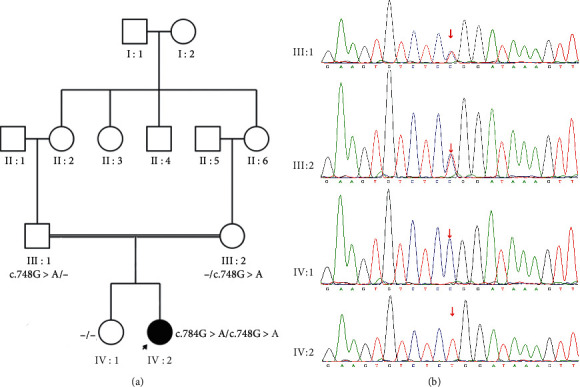
The pedigree and DNA sequencing of family B (a). The pedigree of family B.(b) III : 1 and III : 2 had a heterozygous c.748G > A variant in the *MKKS* gene, IV : 1 had none of the mutant allele, and proband IV : 2 had a homozygous c.748G > A variant.

WES was performed in six family members (III : 4, III : 5, IV : 1, IV : 5, IV : 8, and IV : 10) of family A. Bioinformatics analysis revealed novel compound heterozygous variants c.235T > G (p.T79P) and c.534 + 1G > T in the *BBS2* gene in family A. Sanger sequencing was performed in all of the available family members, which confirmed that all the affected members had the compound heterozygous variants and the unaffected members had no more than one of the two variants (Figure 1). Variant c.235T > G (p.T79P) was transmitted from the father (III : 4), and variant c.534 + 1G > T was transmitted from the mother (III : 5). Variant c.235T > G causes a substitution of residual 79 in *BBS2* from threonine (Thr) to proline (Pro). Thr79 is a conserved residue among vertebrates (Figure 2(a)). Hydrophilic Thr changes to hydrophobic Pro, causing the protein structure to change (Figure 2(b)). The c.534 + 1G > T variant was predicted to change the splice site by HSF. WES was performed in IV:2 of family B, and a known homozygous variant, the c.748G > A (p.G250R) variant in the *MKKS* gene, was identified [32], which was segregated with BBS in this family and was passed to IV : 2 by each parent. However, the *MKKS* variant was absent in unaffected IV : 1 (Figure 3).

### 3.2. Ocular Findings

**Figure 4:**
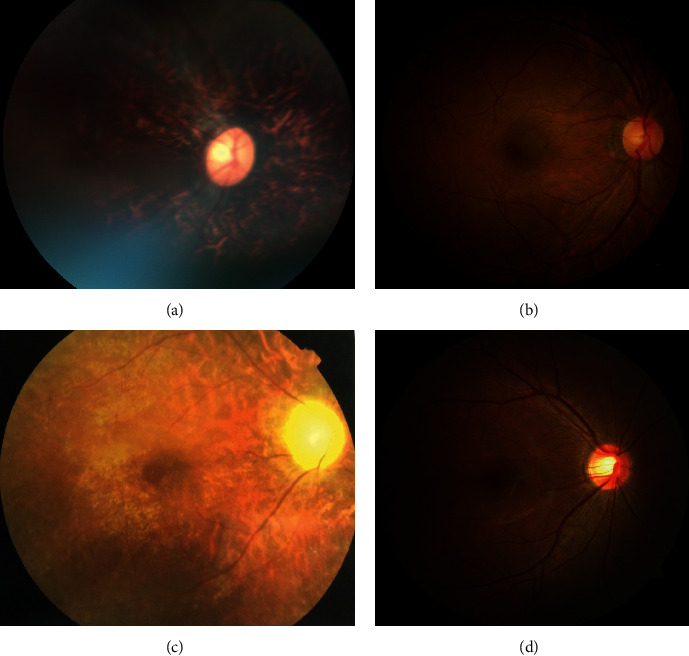
Fundus photos of family A and family B (a). Fundus photo of IV : 2 in family A (affected) showing vascular attenuation, retinal pigment epithelium (PRE) disturbance, and disk pallor. (b) Normal fundus photo of III : 4 in family A (unaffected). (c) Fundus photo of IV : 2 in family B showing vascular attenuation, disc pallor, and retinal pigment epithelium disturbance. (d) Normal fundus photo of IV : 1 in family B.

In family A, IV : 10 was the proband whose poor vision, night blindness, and seeking of medical help brought the entire family to the hospital. After an examination of all of the family members, it was found that all four of those affected by BBS had night blindness, poor visual acuity, and horizontal nystagmus. No light perception was detected in IV : 1 and IV : 2, and finger counting at 1.5 meters for the right eye and at 2.0 meters for the left eye was detected in IV : 3 (because of poor vision and illiteracy, she could not read the visual acuity chart). The electroretinogram of IV : 10 showed undistinguishable photopic and scotopic responses. The ocular findings of this family are summarized in Table 1. The fundus examination showed vascular attenuation as well as disk pallor (Figure 4). Considering the symptoms and ocular examinations, a diagnosis of retinitis pigmentosa was given to this family.

In family B, the proband IV : 2 had decreased vision with night blindness in both eyes since early childhood. Her visual acuity was finger counting at 5 centimeters for the right eye and hand movement at 20 centimeters with horizontal nystagmus. The fundus examination revealed vascular attenuation as well as disc pallor and retinal pigment epithelium changes (Figure 4). The electroretinogram showed dramatically reduced photopic and scotopic responses, confirming the diagnosis of retinitis pigmentosa.

### 3.3. Systemic Clinical Data

**Figure 5:**
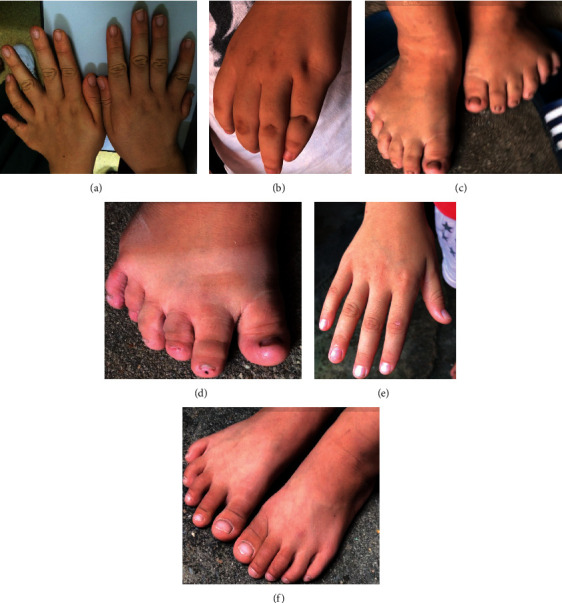
The hands and toes of affected and unaffected participants from family A and family B (a). Hands of IV :2  in family B showing polydactyly. (b) Hand of IV : 2 in family A with surgically treated extra finger. (c) Brachydactyly and polydactyly (six toes) of IV : 2 in family A (d). Brachydactyly and polydactyly (six toes) of IV : 10 in family A (e). (f) Normal hands and toes of IV : 6 in family A.

All of the detailed clinical data are summarized in Table 1. In family A, all the affected participants were overweight with a body mass index (BMI) ranging from 26.5 to 41.9, while the BMI ranged from 15.8 to 16.5 in the unaffected siblings. The blood pressure of III : 1, III : 2, and III : 3 was 132/90 mmHg, 135/95 mmHg, and 184/114 mmHg, respectively (Table 1). All of those affected had polydactyly (six fingers and six toes bilaterally) but were without speech delay and dental anomalies (Figure 4). The extra fingers were surgically removed at birth (Figure 5). IV : 1, IV : 2, and IV : 3 had delays in the onset of secondary sex characteristics and menarche, and genital anomalies were detected by B scan in IV : 1, IV : 2, and IV : 3 with small-sized ovaries and uteruses. IV : 10 had micropenis detected at birth and small-volume testes detected by B scan. Meanwhile, IV : 1, IV : 2, and IV : 3 had poor coordination, IV : 1 and IV : 3 had hyposmia, and IV : 3 had a hearing defect (Table 1). In family B, the proband had polydactyly with a blood pressure of 113/63 mmHg and BMI of 23.9. A blood test revealed normal glucose. B scans of the cardiovascular, urinary, and reproductive systems showed normal function. However, B scan showed the gallbladder polyps and thyroid nodules, and blood test detected high total cholesterol, high low-density lipoprotein cholesterol, high apolipoprotein B, and high uric acid in the proband of family B.

## 4. Discussion

In this study, we report one novel compound heterozygous variant in the *BBS2* gene in a four-generation Chinese family and a known homozygous variant in the *MKKS* gene as well as on the comprehensive ocular manifestations and systemic features.

The primary features of BBS include rod-cone dystrophy, polydactyly, obesity, genital anomalies, renal anomalies, and learning difficulties, and the secondary features include speech delay, developmental delay, diabetes mellitus, dental anomalies, congenital heart disease, brachydactyly or syndactyly, ataxia or poor coordination, and anosmia or hyposmia. However, phenotypes vary according to the different causative genes. In family A, the patient with the compound heterozygous *BBS2* variants had all the primary features except for the renal anomalies, which was confirmed by the B scan. Among the secondary features, the patient had developmental delay, brachydactyly, poor coordination, and anosmia. The clinical features within the family were exactly the same. However, the patient with the homozygous *MKKS* variants presented ocular abnormalities with polydactyly and without other systemic features. The phenotype variations between genes have been illustrated in previous studies. Carmi et al. illustrated differences in the limb distribution of postaxial polydactyly and the extent and age-association of BMI among patients mapped to loci *BBS2*, *BBS3*, and *BBS4* [33]. Ullah et al. presented the differences in the location of polydactyly, cognitive impairment, renal impairment, and syndactyly in patients with variants in *BBS7*, *BBS8*, *BBS10*, and *MKKS* [34]. Within the same family, the most common variation was in limb distribution [34]. However, retinitis pigmentosa was detected constantly in all affected subjects with variants in BBS genes [34, 35]. In a previous study of a patient with the c.748G > A (p.G250R) variant in the *MKKS* gene, the patient presented with obesity and mental delay [32]; however, the phenotype of the patient in this study with the same *MKKS* variant is different. Further studies are needed to illustrate the variabilities in phenotypes even in patients with the same variant in the *MKKS* gene.

Referred to the diagnostic criteria of BBS, clinical diagnosis is made by the presence of either four major features or three major features and two minor features [36]. Family A met four major features (rod-cone dystrophy, polydactyly, obesity, and genital anomalies); however, family B only met two major features (rod-cone dystrophy and polydactyly). Nonetheless, the proband of family B presented with the gastrointestinal and endocrine/metabolic abnormalities, which were considered as minor features of BBS [35]. Forsyth and Gunay-Aygan pointed out the limitation of the clinical criteria that many of these clinical features emerge throughout infancy, childhood, and young adulthood, and for the individuals who are considered, the diagnosis of BBS, periodically review is needed [35]. Phenotypic spectrum of disease due to genetic variation should be taken into account, rather than just the clinical diagnosis itself.

Several studies have reported on the triallelic inheritance [30, 37] of BBS genes, where a patient with a homozygous R315W variant of the *BBS2* gene was also homozygous by descent for the *BBS4* locus [37], and a patient with a homozygous D104A variant of the *BBS2* gene also had a homozygous R632P variant in the *BBS1* gene [37]. The third variant of the triallelic inheritance was considered to have a modifying effect that causes the phenotypic diversity [38]. However, in family A of the current study, WES was performed and only compound heterozygous variants was detected in the *BBS2* gene, and no variants were detected in other BBS-associated genes except for single nucleotide polymorphism with high frequency.

This study presented one family with two novel *BBS2* variants, expanding the variant spectrum of BBS, and one family with a known homozygous *MKKS* variant. The phenotypic similarity in family A with the *BBS2* variant, and the phenotypic difference between family B and the family in the previous study with the same *MKKS* variant, will contribute to improved understanding of BBS.

## Figures and Tables

**Table 1:** Clinical features of families with BBS.

Family	ID	Gene	DNA change	G	Age	Eye	UCVA		RCD	Other	P	GA	RA	Obesity			BP	HR	SD	DA	BS	AP	AH	HD
					(yrs.)	NB	OD	OS						H (cm)	W (Kg)	BMI	(mm Hg)							
A	II : 4	BBS2	c.[=]; [=]	M	67	N	6/6	6/6	N	N	N	N	N	NA	NA	NA	NA	NA	N	N	N	N	N	N
A	II : 5	BBS2	c.[=]; [534 + 1G > T]	F	60	N	NA	NA	N	N	N	N	N	162	61.9	23.6	123/69	92	N	N	N	N	N	N
A	II : 3	BBS2	c.[=]; [=]	F	62	N	6/6	6/7.5	N	N	N	N	N	166	85.2	30.9	139/83	73	N	N	N	N	N	N
A	III : 2	BBS2	c.[235T > G]; [=]	M	57	N	6/4.8	6/4.8	N	N	N	N	N	170	70.0	24.2	129/84	72	N	N	N	N	N	N
A	III : 4	BBS2	c.[235T > G]; [=]	M	43	N	6/4.8	6/4.8	N	N	N	N	N	170	76.4	26.4	124/78	93	N	N	N	N	N	N
A	III : 6†	BBS2	c.[=]; [=]	F	42	N	6/60	6/60	N	HM	N	N	N	162	64.5	24.6	106/70	71	N	N	N	N	N	N
A	III : 5‡	BBS2	c.[=]; [534 + 1G > T]	F	39	N	6/60	6/60	N	HM	N	N	N	158	69.7	27.9	120/81	81	N	N	N	N	N	N
A	III : 3	BBS2	c.[=]; [=]	F	51	N	6/4.8	6/4.8	N	N	N	N	N	156	40.6	16.7	114/71	95	N	N	N	N	N	N
A	IV : 1	BBS2	c.[235T > *G*]; [534 + 1G > T]	F	20	Y	NLP	NLP	Y	Nys	Y	Y	N	160	71.7	28.0	132/90	95	N	N	Y	Y	Y	N
A	IV : 2	BBS2	c.[235T > G]; [534 + 1G > *T*]	F	18	Y	NLP	NLP	Y	Nys	Y	Y	N	157	103.4	41.9	135/95	119	N	N	Y	Y	NA	N
A	IV : 3	BBS2	c.[235T > G]; [534 + 1G > T]	F	16	Y	FC/1.5m	FC/2m	Y	Nys	Y	Y	N	167	84.1	30.2	184/114	94	N	N	Y	Y	Y	Y
A	IV : 5	BBS2	c.[=]; [534 + 1G > T]	M	11	N	6/4.8	6/6	N	N	N	N	N	144	34.1	16.4	103/73	91	N	N	N	N	N	N
A	IV : 6	BBS2	c.[=]; [534 + 1G > T]	F	9	N	6/3.8	6/6	N	N	N	N	N	129	26.4	15.9	110/68	86	N	N	N	N	N	N
A	IV : 7	BBS2	c.[235T > G]; [=]	F	7	N	6/4.8	6/4.8	N	N	N	N	N	113	20.4	16.0	NA	NA	N	N	N	N	N	N
A	IV : 8	BBS2	c.[=]; [534 + 1G > T]	F	5	N	6/7.5	6/9.5	N	N	N	N	N	108	18.4	15.8	NA	NA	N	Y	N	N	N	N
A	IV : 9	BBS2	c.[=]; [=]	F	3	N	NA	NA	N	NA	N	N	N	90	13.4	16.5	NA	NA	N	N	N	N	N	N
A	IV : 10	BBS2	c.[235T > G]; [534 + 1G > *T*]	M	2	Y	NA	NA	Y	Nys	Y	Y	N	82	17.8	26.5	NA	NA	Y	N	Y	NA	NA	NA
A	IV : 11	BBS2	c.[235T > G]; [=]	M	25	N	6/4.8	6/6	N	N	N	N	N	173	66.3	22.2	122/82	65	N	N	N	N	N	N
A	IV : 12	BBS2	c.[235T > G]; [=]	F	23	N	6/6	6/6	N	N	N	N	N	157	40.2	16.3	107/75	89	N	N	N	N	N	N
B	IV : 2	MKKS	c.[748G > A]; [748G > A]	F	19	Y	FC/5cm	HM/20cm	Y	Nys	Y	N	N	155	57.5	23.9	111/63	80	N	N	N	N	N	N

G, gender; NB, night blindness; UCVA, uncorrected visual acuity; BP, blood pressure; HR, heart rate; BMI, body mass index; Y, yes; N, No; NA, not available; RCD, rod-cone dystrophy; † and ‡: the III:6 and III:5 are with high myopia, the visual acuity was not corrected by spectacles; M, male; F, female; No, normal; Age, age at examination; OD, right eye; OS, left eye; HM, high myopia; NLP, no light perception; Nys, nystagmus; P, polydactyly; GA, genital anomalies; RA, renal anomalies; H, height; W, weight; SD, speech delay; DM, diabetes mellitus; DA, dental anomalies; BS, brachydactyly/ syndactyly; AP, ataxia/poor coordination; AH, anosmia/hyposmia; HD; hearing defect. Yrs., years old.

## Data Availability

The data that support the findings of this study are available from the corresponding author (Xiaoyan Ding) upon reasonable request.
